# A Novel Text-Mining Approach for Retrieving Pharmacogenomics Associations From the Literature

**DOI:** 10.3389/fphar.2020.602030

**Published:** 2020-11-10

**Authors:** Maria-Theodora Pandi, Peter J. van der Spek, Maria Koromina, George P. Patrinos

**Affiliations:** ^1^ Laboratory of Pharmacogenomics and Individualized Therapy, Department of Pharmacy, School of Health Sciences, University of Patras, Patras, Greece; ^2^ Erasmus University Medical Center, Faculty of Medicine and Health Sciences, Department of Pathology, Bioinformatics Unit, Rotterdam, Netherlands; ^3^ Department of Pathology, College of Medicine and Health Sciences, United Arab Emirates University, Al-Ain, United Arab Emirates; ^4^ Zayed Center of Health Sciences, United Arab Emirates University, Al-Ain, United Arab Emirates

**Keywords:** text mining, natural language processing, Pubtator, Pubmed, pharmacogenomics associations, FastText, biomedical text classification, supervised learning

## Abstract

Text mining in biomedical literature is an emerging field which has already been shown to have a variety of implementations in many research areas, including genetics, personalized medicine, and pharmacogenomics. In this study, we describe a novel text-mining approach for the extraction of pharmacogenomics associations. The code that was used toward this end was implemented using R programming language, either through custom scripts, where needed, or through utilizing functions from existing libraries. Articles (abstracts or full texts) that correspond to a specified query were extracted from PubMed, while concept annotations were derived by PubTator Central. Terms that denote a Mutation or a Gene as well as Chemical compound terms corresponding to drug compounds were normalized and the sentences containing the aforementioned terms were filtered and preprocessed to create appropriate training sets. Finally, after training and adequate hyperparameter tuning, four text classifiers were created and evaluated (FastText, Linear kernel SVMs, XGBoost, Lasso, and Elastic-Net Regularized Generalized Linear Models) with regard to their performance in identifying pharmacogenomics associations. Although further improvements are essential toward proper implementation of this text-mining approach in the clinical practice, our study stands as a comprehensive, simplified, and up-to-date approach for the identification and assessment of research articles enriched in clinically relevant pharmacogenomics relationships. Furthermore, this work highlights a series of challenges concerning the effective application of text mining in biomedical literature, whose resolution could substantially contribute to the further development of this field.

## Introduction

Over the span of 10 years, technological achievements and advances have shifted the direction of pharmacogenomics (PGx) research from candidate gene PGx to large-scale PGx studies (Giacomini et al., 2017; Lavertu et al., 2018). Therefore, the identification and delineation of pharmacogenomics relationships are crucial for the improvement of the fields of PGx and personalized medicine. However, elucidating the role of specific genes in drug response and toxicity requires years of research and—in most cases—manual curation of thousands of articles. To this end, biomedical text mining can be proven useful in reducing manual efforts of curating important PGx relationships from the available literature.

As previously shown, text mining has become a widely used approach for the identification and extraction of information from unstructured text (Westergaard et al., 2018). More precisely, text mining is used to extract facts and relationships in a structured form, which can then be used for a variety of implementations, such as database annotation or delineation of complex relationships and transferring of useful knowledge between different research domains (Renganathan, 2017). In terms of biomedical text mining, PubMed is primarily implemented for this purpose, owing to the easy and fast extraction of information regarding biological entities, such as genes and proteins.

Guin et al. (2019) proposed a semiautomated text-mining approach to retrieve a complete pharmacogenomics (PGx) resource integrating disease–drug–gene polymorphism relationships and thus further improving the field of precision medicine. Their results were subsequently validated by assessing the performance (precision = 0.806) with benchmark datasets like Pharmacogenomic Knowledgebase (PharmGKB), Online Mendelian Inheritance in Man (OMIM), and the Comparative Toxicogenomics Database (CTD) and further comparing the retrieved associations with 362 commercially used US Food and Drug Administration (FDA)-approved drug labeling biomarkers. In another study, Lever et al. (2020) presented the PGxMine resource, which is a text-mining resource of pharmacogenomic associations from all accessible published literature to assist in the curation of PharmGKB. More specifically, Lever developed a supervised machine learning pipeline to extract associations between a genetic variant or haplotype and a chemical. PGxmine resource was further evaluated by PharmGKB curators, in order to demonstrate its efficacy as a pharmacogenomics text-mining tool.

Up to submission of this study, there has been a limited number of published papers, in which biomedical text-mining methodologies have been implemented in order to not only retrieve important disease/drug-gene/polymorphism relationships but also to assess the Sensitivity and Specificity of these relationships. Moreover, most of the existing databases (i.e., OMIM, Human Gene Mutation Database (HGMD), CTD, and PharmGKB) which curate pharmacogenomics relationships employ manual curation of biomedical literature in order to exploit disease or drug-related genetic association relationships in humans. Consequently, much of this information remains inaccessible in the unstructured text of biomedical publications. This observation further demonstrates the need for an accurate and automated process which will highlight important and clinically relevant PGx relationships.

In this study, we propose a novel biomedical text-mining system, which retrieves clinically relevant biomedical information, while also comparing the accuracy of the retrieved information with data extracted from PharmGKB. Text-mining annotation is also performed not only for PubMed abstracts but also for full-text articles. The key feature of this study is the use of advanced text mining and natural language processing (NLP) to tabulate the most important and clinically relevant pharmacogenomics relationships by comparing these to gold standard datasets, thus further demonstrating their potential clinically utility.

## Materials and Methods

**FIGURE 1:**
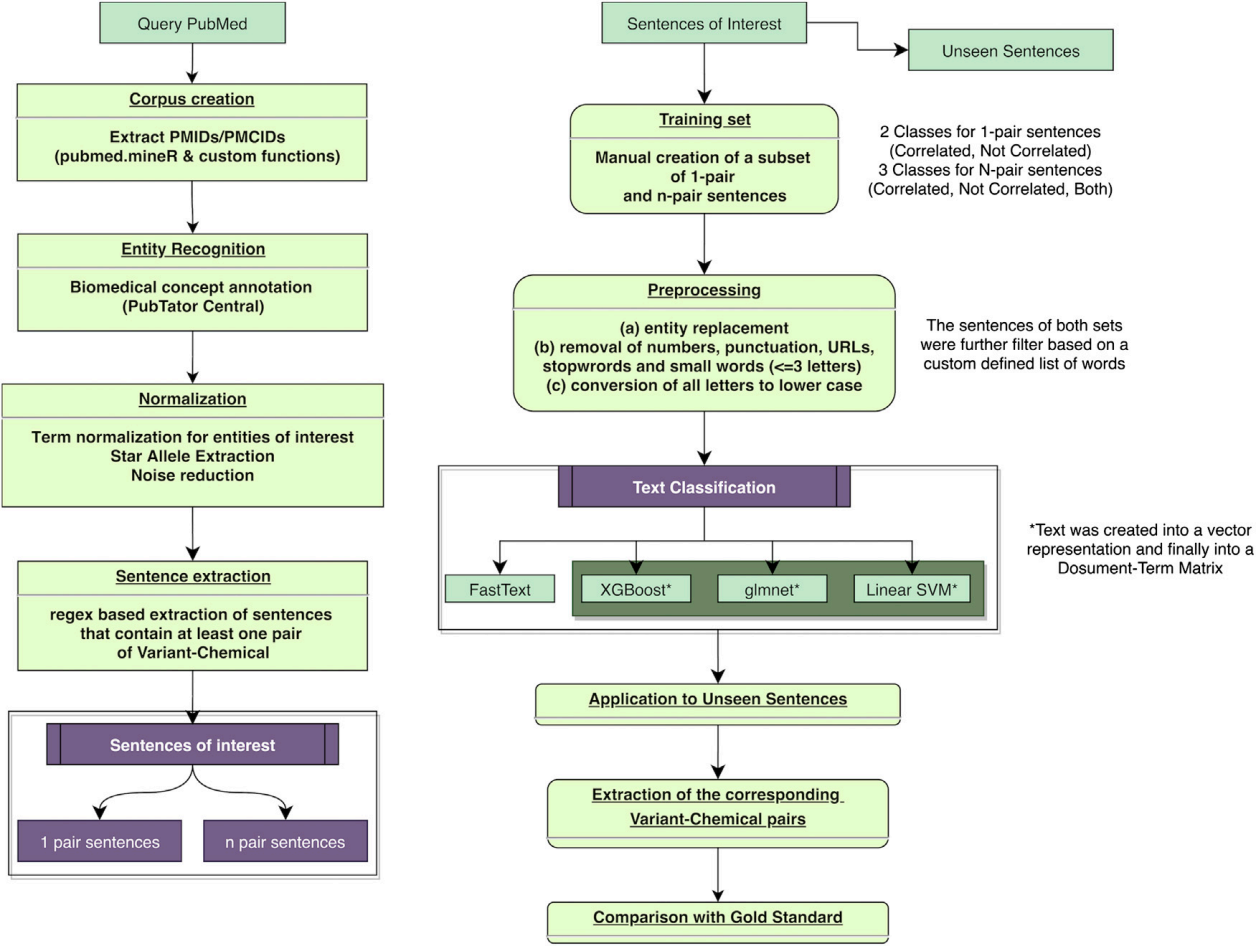
Flowchart of the proposed automated text-mining approach and the validation steps for the retrieved literature relationships. PMIDs, PubMed identifiers; PMCIDs, PubMed Central identifiers (attributed to full-text articles only).

The present biomedical text-mining approach includes the following common steps of natural language processing (NLP): Figure 1 corpus creation, concept annotation and normalization, identification, extraction and filtering of sentences of interest and finally, and text classification with the purpose of discovering pharmacogenomics associations. The derived associations were subsequently compared with gold standard dataset from PharmGKB. The entire project was created using custom code and available packages in R programming language (version 4.0.0) (R Development Core Team, 2020).

### Corpus Creation

The first step was the collection of published literature that is likely to contain information about pharmacogenomics associations, relevant to human species but not review articles. These prerequisites were summed up in the following query: ‘(pharmacogen*[Text Word] AND (“humans” [MeSH Terms]) NOT (Review [ptyp]))’.

Querying the NCBI’s PubMed database (via API, easyPubMed R package) in May 2020 resulted in the extraction of 11,302 PMIDs (standard identifiers for articles present in PubMed). Those PMIDs were consequently converted into their corresponding PubMed Central Identifiers or PMCIDs (i.e., standard identifiers for articles freely available as full text in the PubMed Central database), by using NCBI’s ID converter. Out of the 11,302 originally retrieved articles, only the 3,165 were freely available, while for the rest, we could access only the title and the abstract.

### Concept Annotation

Concept annotation of the collected corpus with regard to biological entities (i.e., genes/proteins, genetic variants, diseases, chemicals, species, and cell lines) was performed with a custom function, by programmatically accessing, through RESTful API and PubTator Central (PTC) (Wei et al., 2019). To this end, the information existing in PTC for a given article was retrieved in BioC-JSON format and processed in order to extract the relevant annotation from each text passage, either from the title and abstract of a paper or from the entire text, depending on the availability of a PMID or a PMCID, respectively. Basic information regarding the article (PMID, PMCID, journal, year, and authors), as well as information regarding each passage (Type, Section, Offset, and Text) and the annotated terms identified in this specific passage (Type, Offset, Text, and Identifier) was extracted. More specifically, PTC provides for annotated genes or proteins their corresponding NCBI Gene identifiers, while variants are accompanied by their dbSNP rs identifiers. Furthermore, identified diseases and chemicals are characterized by their normalized MeSH identifiers. Those PTC-annotated articles were filtered to keep only those that contained at least one biomedical concept corresponding to Chemical and at least one concept corresponding to Gene or Mutation. In addition, papers that do not contain a Species concept with NCBI Gene ID 9606 (*Homo sapiens*) are filtered out. Finally, a data frame aggregating the results was created, and any irrelevant annotations were removed in order to retain only terms belonging to one of the following categories: Gene, Mutation, and Chemical.

### Concept Normalization

Since the results from PTC could potentially contain a significant amount of task-irrelevant terms, each category of Concepts of interest (Genes, Mutations, and Chemicals) was further evaluated, while only terms identified in the abstract or in paragraphs of an article were maintained (i.e., filtering out terms found in tables, titles, and references). Regarding Genes, only those corresponding to a human gene were kept, while entries corresponding to the pattern “*p* = .,” which was mistakenly annotated as Gene, were discarded. From Mutations, only those accompanied by a dbSNP rsID identifier were kept. Normalizing Chemicals was proven to be one of the most challenging tasks. The main objectives were to remove as much noise as possible (i.e., remove as many nondrugs) and map the provided identifier to one that could be used to directly compare with the gold standard. As mentioned previously, PTC provides the MeSH identifier of a Chemical found in an article, while PharmGKB provides MeSH IDs only for a limited number of its entries. In addition, some of the PTC provided identifiers are outdated and do not correspond to a current MeSH record. To avoid inconsistencies, a mapping file was provided to us from the team of PTC, converting those outdated IDs into their up-to-date version (as found in MeSH database).

Finally, for the mapping of the provided Chemical MeSH IDs to PharmGKB IDs, the Chemical Vocabulary from Comparative Toxicogenomics Database (CTD) was accessed on April 10, 2020. This vocabulary contains up-to-date MeSH IDs, is a subset of MeSH’s dataset, after the exclusion of entries that are not molecular reagents, environmental chemicals, or clinical drugs, and also provides a list of DrugBank IDs, which can be used to connect to PharmGKB IDs. In order to keep mostly those that are actually drugs and eliminate any remaining noise, the data from CTD and PharmGKB’s chemical. tsv (downloaded on April 10, 2020) were combined based on the provided DrugBank ID (a common key between the two datasets) and were subsequently filtered to keep only the following Chemical Types (as defined in PharmGKB): Drug, Drug/Biological Intermediate, Prodrug, Drug/Ion, and Drug/Metabolite, leading to a list of 1,449 chemicals. This list was further manually curated, leaving 1,395 chemicals, and was used to remove “nondrug” chemicals from our data frame. Finally, the remaining Chemical IDs were queried to MeSH (via NCBI’s e-utilities) to get the corresponding MeSH terms for these compounds.

### Star Allele Extraction

One of the limitations of the tools used by PTC for the identification of biomedical entities is their inability to distinguish Star Alleles, which are of utmost importance in pharmacogenomics and their misclassification as Genes. To overcome this obstacle, a search, based on regular expressions, was performed on the texts that contained a term characterized by PTC as “Gene,” to identify those complying with the Star Allele nomenclature. This process was applied solely on cases of terms regarding Genes that are already known to have Star Alleles (based on entries of PharmVar and the genes mentioned by Lee et al. (2019)). String matching for each of those genes was performed to identify potential Star Alleles within the corpus.

### Sentence Extraction

Subsequently, the sentences that contained a concept of interest (Mutation, StarAllele, or Chemical) were extracted from the corresponding paragraphs based on string matching and the provided coordinates of each term within this paragraph. Further filtering of the sentences led to a subset of sentences containing at least one Mutation or StarAllele (which we both included under the term “Variant”) and at least one Chemical compound. As expected, some of the derived sentences contain one pair of Variant-Chemical (1-pair sentences), while others might contain multiple mentions of Variant, Chemicals, or both, leading to multiple Variant-Chemical pairs (n-pair sentences). Although these two pairs of sentences received similarly preprocessing, they were treated differently during classification and training set creation.

### Relation Extraction

In order to extract the existing relationships (if any) between the Variants and the Chemicals present in each sentence, a subset from each sentence category (1-pair and n-pair) was manually curated and two distinct training sets were created. Consequently, those training sets were used to train four different classification algorithms and the models created were applied to the remaining, unseen, sentences and results were finally compared with a PharmGKB-derived Gold Standard set of Variant-Chemical pairs.

### Training Set Creation

Regarding sentences that discuss only one pair of Variant-Chemical, a sentence was classified as “Correlated,” if the context of that sentence implied a clear association between the pair of Chemical-Variant in question or as “Not Correlated” if the context of the sentence implied no association, unclear associations, conflicting results, or indirect associations. Therefore, the Variant-Chemical pair’s class (Correlated or Not Correlated) determines the class of the sentence that contains it. In the case of n-pair sentences, each possible pair of Variant-Chemical for a sentence was individually assessed, and the results were aggregated to create three classes. When all the pairs mentioned in a sentence were classified as “Correlated,” that sentence received the same classification (“Correlated”). The same logic applies for n-pair sentences where all the pairs were classified as “Not Correlated” (those sentences were also classified as “Not Correlated”). Finally, n-pair sentences that contained pairs classified as “Correlated” as well as pairs classified as “Not Correlated” were put under a new class (named “Both”). The training set for 1-pair sentences consists of 1,039 sentences (and a corresponding number of Variant-Chemical pairs), while the n-pair sentences training set consists of 600 distinct sentences, containing 1,880 Variant-Chemical pairs.

### Sentence Preprocessing

Since biomedical texts might contain a wide variety of words, numbers, URLs and links, names of genes, chemicals, diseases, species, and so on, as well as abbreviations or the names of the writers of different articles which might be discussed, and all of these might add noise, rather than aiding the classification task; the derived sentences were preprocessed before being used to train an algorithm, as well as before a trained model was applied to them. As a first step and since the specific variants and chemicals found in a sentence were not of particular importance, the corresponding Variants found in a sentence of interest were replaced using string matching by the term “ClassVariant,” while Chemicals were replaced by the term “ClassChemical.” As a next step, all the words were converted to lowercase while URLs, numbers, and punctuation were removed from a sentence. Additionally, words with three characters or less (with the exception of “no,” “not,” “nor,” “ae,” “aes,” and “adr”) were removed. Finally, a set of custom stopwords was removed in order to reduce the number of unique evaluated words. This set of stopwords consists of PubMed’s stopwords, after minor modifications (https://www.ncbi.nlm.nih.gov/books/NBK3827/table/pubmedhelp.T.stopwords/); gene names based on Human Gene Nomenclature Committee (HGNC), after being converted to lowercase and having numbers and punctuation removed from them (to match the preprocessing that has already been applied to the words if a sentence); and finally frequent words common in all the classes. These processed sentences that were created were finally filtered to maintain only those containing at least one word of interest, as available in the Supplemental Material, since it was noted that this step aids toward improving the Precision of the created classifiers.

### Classification Task

Four algorithms were used to create text classification models for the purpose of identifying PGx-related relations (FastText, Linear kernel SVM, XGBoost, and Lasso and Elastic-Net Regularized Generalized Linear Models). FastText is an open-source and free library, written in C++, which performs both supervised (classification) and unsupervized (word representation) tasks regarding text, while at the same time supports multiprocessing during training. Although a linear classifier (multinomial logistic regression), it is proven to be efficient and comparable with deep learning classifiers in many tasks. In a nutshell, the initial word embeddings are averaged to create a sentence vector which is then used to train the linear model (Joulin et al., 2017). This algorithm was implemented using fastRtext R library, a wrapper for FastText C++ code from Facebook (Benesty, 2019).

The hyperparameter tuning, training, and the computation of the corresponding performance metrics (for all four models) was performed; caret R library (Kuhn, 2020) was used. For Linear kernel Support Vector Machines (SVM), the implementation found in the kernlab R package was used, while for Lasso and Elastic-Net Regularized Generalized Linear Models (Friedman et al., 2010), the original library used through caret was glmnet. Finally, for XGBoost, an ensemble classifier (gradient boosting decision trees), the original implementation of the algorithm is described by Chen and Guestrin (2016). For these three algorithms, the sentences were beforehand converted into sparse Document-Term Matrices (DTM), with the help of text2vec R package (Dmitriy Selivanov and Wang, 2020). More specifically, each sentence is tokenized into word tokens and a vocabulary consisting of the unique words is created. This vocabulary was pruned to contain only words which occur between 3 and 300 times in the training corpus for the 1-pair sentences and from 3 to up to 400 times for n-pair the sentences and was consequently used to create the different sentence vectors. Finally, a DTM is created independently for the sentences constituting the training and testing set, respectively.

The task of classification was approached independently for those two cases, thus leading to the training of eight models: four binary classifiers for 1-pair sentences and four multiclass classifiers for n-pair sentences. In order to assess the performance of those models, 10-fold Cross Validation was performed for the algorithms trained with the 1-pair sentences, while 5-fold Cross Validation was chosen for models trained with the smaller set of n-pair sentences. The optimal parameters are presented in Table 1 for FastText and Table 2 for the three remaining algorithms.

**TABLE 1:** Presentation of the default and selected hyperparameter values for FastText algorithm.

Hyperparameter	Default value	Values used for both 1-pair and n-pair sentences
Size of vector (dim)	100	200
Minimal number occurrences of a word (minCount)	1	5
Size of the context window (ws)	5	2
Learning rate (lr)	0.1	0.1
Number of epochs (epoch)	5	50
Maximum length of a word ngram (worNgrams)	1	2
Loss function (loss)	Softmax	ns (negative sampling)

**TABLE 2:** Presentation of the default and selected, after grid search, hyperparameter values SVM, XGBoost, and glmnet models.

Model	Hyperparameters	Default values	1-Pair	n-Pairs
Linear SVM	Cost (C)	1	1	1
XGBoost	Learning rate (eta)	0.3 [0, 1]	0.2	0.2
	Maximum depth of a tree [maxdepth]	6 [0, ∞)	4	6
	Subsample ratio of the training instances (subsample)	1 (0, 1]	0.7	0.7
	Number of boosting iterations (nrounds)	—	50	50
glmnet	Mixing percentage (alpha)	1 [0, 1]	0.1	1 (lasso penalty)
	Regularization parameter (lambda)	—	0.02888342	0.02229455

### Classification Metrics

Detailed explanations regarding the computation of performance metrics for binary classification are found in Supplementary Tables S1, S2. With regard to the multiclass classification task for n-pair sentences, the “one-vs.-all” approach was followed for the estimation of TP, TN, FP, and FN outcomes and the computation of the corresponding performance metrics.

### Application to the Rest of the Corpus

The derived text classifiers were consequently applied in the remaining un-curated literature (unseen sentences), and their performance was evaluated based on the gold standard dataset from PharmGKB. Before the application of the models, the “testing” sentences were preprocessed in a similar manner (see *Sentence Preprocessing*) as the “training” sentences that were used to train the corresponding algorithms.

### Comparison With PharmGKB Gold Standard Dataset

The extracted pharmacogenomics relationships were further verified by using a PharmGKB gold standard dataset, consisting of manually curated pairs of variants (Mutations or Star Alleles) and chemicals, for which the annotation evidence was annotated as “associated” (and which constitute the Positive pairs) or either as “nonassociated” or as “ambiguous” (Negative pairs). The computation of performance metrics is based on the determination of True Positive (TP), True Negative (TN), False Positive (FP), and False Negative (FN) outcomes and the computation of Accuracy, Sensitivity/Recall, Specificity, Precision/Positive Predictive Value, and Negative Predictive Value, as it is described in Supplementary Table S2.

## Results

### Overall Synopsis

11,302 published articles, available in PubMed, were extracted based on the custom query, as described in the *Methods* section, while only the 28% of them (3,165) were freely available as full texts (Table 3). The identification of biomedical entities of interest via PubTator Central resulted in 5,307 papers with unique PMIDs (2,257 or 42.5% of which are available as full texts). The remaining articles (5,995) were either not annotated by PTC as of submission of the study, or they did not meet the criteria to be used in the following text-mining steps.

**TABLE 3:** Total number of the 1) initially retrieved, 2) annotated by Pubtator Central, and 3) filtered papers, based on the “pharmacogenomics-related” Pubmed query, as described in the *Methods* section.

Papers resulting from query	11,302 unique PMIDs (3,165 with PMCID)
PTC-annotated papers	5,307 unique PMIDs (2,257 with PMCID)
PTC annotations	Chemicals: 187,850 (5,580 unique) genes: 230,159 (8,853 unique) mutations: 63,855 (13,610 unique) species: 115,520 (433 unique) strains: 54 (9 unique)
Normalized terms	Genes: 5,463 remained chemicals: 805 remained mutations: 5,467 remained
Star alleles^a^	11,201 entries (mistaken as gene entries)
Sentences of interest	With 1 pair: 3,574 with multiple pairs: 1987 (distinct sentences)

PMIDs, PubMed identifiers; PMCIDs, PubMed Central identifiers.

a The number of “not unique” Star Alleles, since some of these are present in multiple copies. This number reflects the amount of Gene mentions that were actually Star Alleles.

The terms, as extracted by PTC, contain a significant amount of noise that could negatively affect the performance of the trained classifiers. Therefore, each category of Concepts of interest (Genes, Mutations, and Chemicals) was further evaluated, to keep only the most relevant terms. After performing Star Allele identification and sentence extraction, a subset of those sentences was manually curated to create training instances that would be used in the relation extraction step. In order to reduce noise added by redundant or very rare words, whose classification value is expected to be limited, each sentence was converted into a vector that contains its most representative words.

### Classification Results

The best hyperparameters for FastText were determined through testing different values for specific parameters and after following thoroughly the provided guidelines (https://fasttext.cc/docs/en/supervised-tutorial.html). In contrast, the best hyperparameters for SVMs, XGBoost, and glmnet were determined through grid search, as the hyperparameter values that maximized the Accuracy metric, calculated using 10-fold Cross Validation.

**FIGURE 2:**
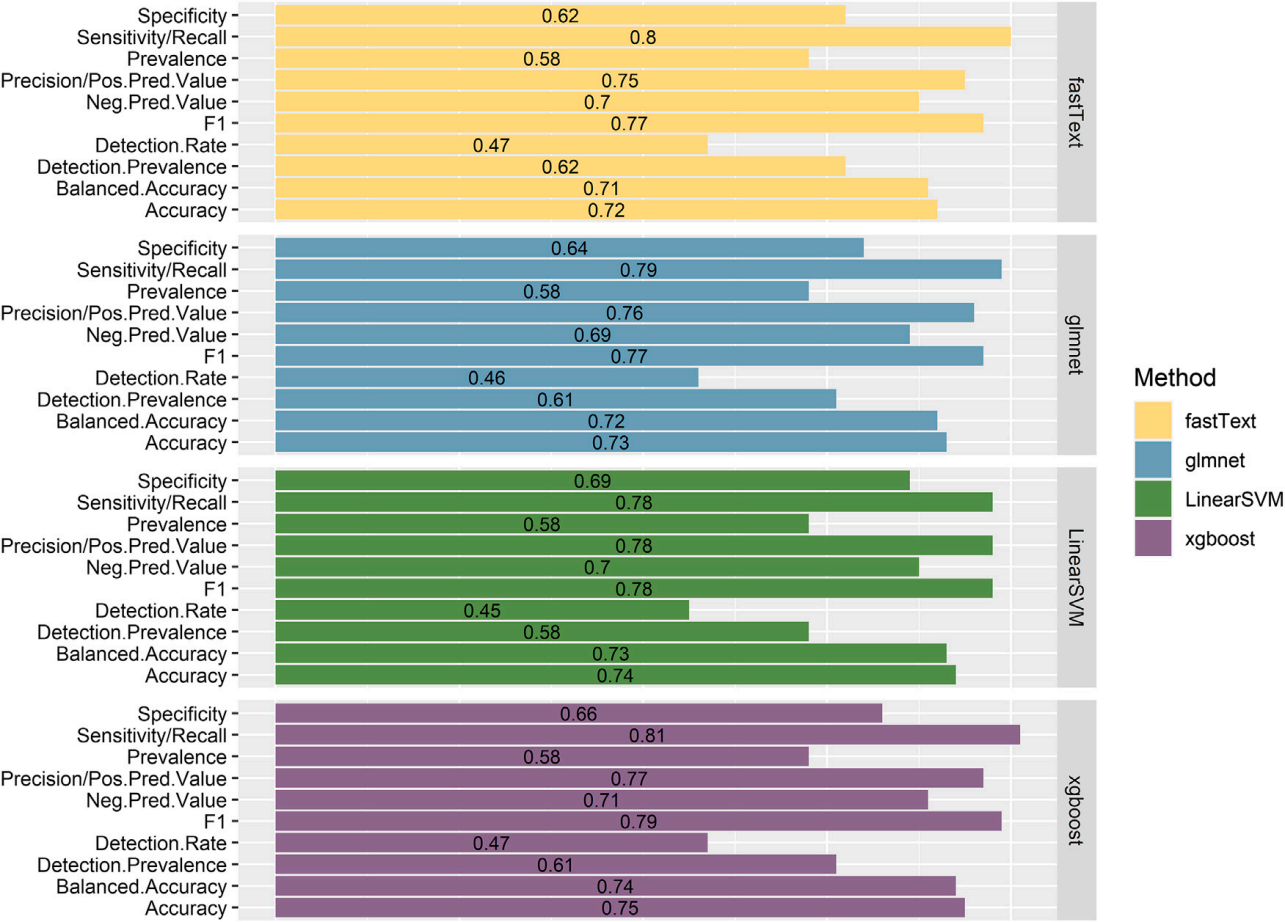
Presentation of the performance metrics, as calculated after using 10-fold Cross Validation with the training data, for all four models trained with sentences discussing one pair of Variant-Chemical (1-pair sentences).

**FIGURE 3:**
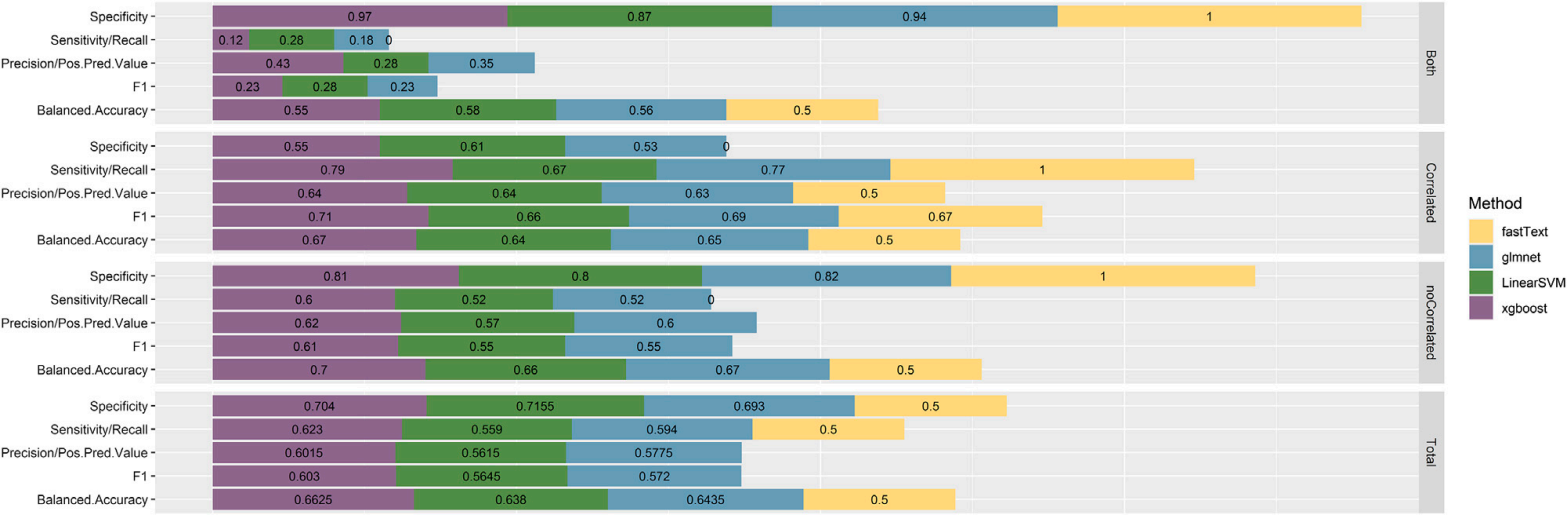
Performance metrics, as calculated after using 10-fold Cross Validation with the training data, for all four models trained with sentences discussing multiple Variant-Chemical pairs (n-pair sentences). The resulting metrics are presented by model and by class, since this is a multiclass classification task, while finally, the by-class metrics for each model separately are weighted with the corresponding class prevalence and summed up to calculate the overall performance metrics.

The performance of the resulting classifiers was evaluated by computing the averaged classification metrics, after performing 10-fold Cross Validation. With regard to classifiers trained using the 1-pair sentences, we can observe (Figure 2) that all the models perform in a similar manner, with XGBoost being the one slightly preceding according to Accuracy, Balanced Accuracy, F1, Sensitivity, Specificity, and Precision. Overall, the performance on this task proves better than expected, considering that no particularly elaborate preprocessing technique was applied to the training data. On the contrary, classifiers created from the n-pair sentences proved to be substantially less effective toward the task they were trained for (Figure 3). This is not surprising, considering the complexity of the sentences and the assumptions made during this step. FastText appears to be more sensitive toward the “Correlated” sentences (Sensitivity = 1), followed by XGBoost (0.79) and glmnet (0.77). However, FastText’s performance regarding the rest class (according to Sensitivity, Precision, Balanced Accuracy, and F1-score) renders this model nonacceptable. For the rest of the algorithms, although they are more effective in identifying “Not Correlated” sentences than FastText, they still perform poorly toward “Both” sentences (Figure 3). Overall, considering all present metrics, XGBoost appears to be the most effective model trained using n-pair sentences, followed closely by the SVM and glmnet models. 

### Validation With a Gold Standard Dataset From PharmGKB

Owing to the poor performance of the classifiers regarding the n-pair sentences, only those trained with 1-pair sentences were compared with the PharmGKB gold standard. Since those sentences focus only on one pair of Variant-Chemical, the Class attributed to a sentence by a classifier is also the one attributed to the candidate pair. However, one pair might appear in different sentences in which the Class might differ (e.g., as the result of conflicting findings in different studies). Consequently, pairs that were classified both as “Correlated” (Positive) and as “Not Correlated” (Negative) in different sentences were considered to belong only to the “Not Correlated” category. Furthermore, since the pairs appearing in the PharmGKB were extracted after manual curation of the published literature, we filtered the gold standard set to keep only Variant-Chemical pairs derived from the same articles (based on PMID), as the ones comprising the set of sentences to which the classifiers were applied. More precisely, True Positive values have described the instances that are classified as “Correlated” and are found in the Positive pairs of the gold standard; True Negative pairs are those classified as “Not Correlated” and found in the Negative pairs of the gold standard; False Positive pairs are the pairs classified as “Correlated” and found in the Negative pairs of the gold standard, while False Negative pairs are the pairs classified as “Not Correlated” and which are found in the Positive pairs of the gold standard. Initially, the gold standard consists of 10,121 curated pairs of Variant-Chemical which after filtering with PMID were reduced to 1,578 (1,337 of which belong to the “Correlated” class and 241 to the “Not Correlated”) (Table 4). Of these, only 104 are found in our test set (53 “Correlated” and 51 “Not Correlated”).

**TABLE 4:** Results stemming from the comparison of the classification results of the four models trained with 1-pair sentences compared with a gold standard dataset, extracted from PharmGKB.

Metric	xgboost	svm	Glmnet	Fastrtext
Filtered unseen sentences				
Accuracy	0.577	0.526	0.538	0.526
Sensitivity/recall	0.512	0.465	0.488	0.349
Specificity	0.657	0.6	0.6	0.743
Precision/positive predictive value	0.647	0.588	0.6	0.625
Negative predictive value	0.523	0.477	0.488	0.481
Original unseen sentences				
Accuracy	0.538	0.529	0.577	0.577
Sensitivity/recall	0.415	0.358	0.434	0.264
Specificity	0.666	0.706	0.725	0.902
Precision/positive predictive value	0.564	0.559	0.622	0.737
Negative predictive value	0.523	0.514	0.552	0.541

TP, TN, FP, and FN were calculated by comparing the resulting classification of the unseen pairs with the pairs present in the Gold Standard and the corresponding metrics were calculated as described in *Methods*. This table presents the metrics calculated regarding the unseen 1-pair sentences with and without filtering based on the define list of words that was used to create the define the training sentences.

## Discussion

Personalized and translational medicine aims toward the discovery and integration of basic biological concepts into the clinical routine. The ever-increasing knowledge about the impact of genomics variation in relation to drug response has yielded emerging research fields such as pharmacogenomics. Recent computational advances, including the creation of algorithms, which retrieve literature information about the association of genes or genomics variants with drug response or adverse effects of drugs, are expected to progress alongside genome-guided medicine (Hansen et al., 2009). Guin et al., 2019 used pubmed. mineR, an R text-mining library intended to be utilized toward analyzing abstracts of biomedical articles (Rani et al., 2015), in order to identify articles of interest, which were consequently evaluated and processed to extract drug-gene-variant relationships, based on the cooccurrence of the corresponding terms (Guin et al., 2019). However, pubmed. mineR is restricted on the abstracts, while the function created for this work can also extract PTC annotations from the entire text, when a PMCID is available.

Another interesting work is this of Pharmspresso (Garten and Altman, 2009), a tool created to identify mentions of human genes, polymorphisms, drugs, and diseases, as well as their relationships according to predefined regular expressions-based templates. The main differences of our approach lie within the way the relationships are extracted (ML-based vs. pattern-based) and the method used for collecting a corpus.

Therefore, the present approach can be exploited to generate PGx relationships published for administered medications among different disease phenotypes. Although further work is essential in order to be able to capture an increased number of PharmGKB biomarkers or biomarkers for which FDA guidelines exist, our text-mining approach can be applied to capture a variety of clinically relevant PGx relationships, for which PharmGKB and FDA guidelines already exist.

Our text-mining approach though came not without any limitations. To begin with, the collection of a complete and concise query, free of irrelevant articles, relies heavily on the formulation of the query performed on PubMed. In addition, the majority of the extracted papers are only available as abstracts, thus reducing the available text that can be evaluated and leading to loss of associations. As highlighted previously elsewhere, being able to analyze the entire text of an article can add valuable information (Garten and Altman, 2009; Westergaard et al., 2018). When the extracted articles are annotated (via PTC), we observe that more than half were removed as they either did not contain the desired information, or this information was not available. Furthermore, issues deriving from the entity recognition tools PTC relies on can further affect the quality of the extracted results, especially with regard to chemicals (e.g., depreciated MeSH IDs or misidentified chemicals). However, despite the current limitations, tools like PubTator Central provide tremendous research possibilities in a great variety of tasks, including the one presented here.

Finally, comparing with a PharmGKB gold standard set of Variants and Chemicals, might not be a suitable option in this case. The status of such a pair in PharmGKB is determined after the manual curation and integration of results from a number of articles, some of which might be conflicting with others, regarding a given pair of Variant-Chemical. On the contrary, in this approach, a simplifying assumption was made, characterizing any pairs with conflicting classifications as “Not Correlated,” an assumption that could be potentially mistaken. Furthermore, the number of gold standard’s Variant-Chemical pairs for the same articles (based on PMID) as the ones constituting the unseen sentences is substantially smaller than the number of tested pairs [(Unseen sentences: 795 pairs without filtering of the unseen sentences, or 673 after filtering) vs. (104 pairs without filtering of the unseen sentences, or 78 after filtering)].

Regardless of the current limitations, the present study described an automated text-mining system which extracts database level annotations from PubMed abstracts and full texts. Such approaches will lead to the identification of clinically meaningful relationships in the era of big data analytics. Although manual curation of the relationships may still be needed to a certain extent, text-mining approaches can be particularly useful in the delineation and curation of clinically meaningful relationships, such as PGx associations.

## Data Availability Statement

The data analyzed in this study is subject to the following licenses/restrictions: Data are available per contacting the authors. Requests to access these datasets should be directed to gpatrinos@upatras.gr.

## Author Contributions

M-TP, MK, and GP conceived the study; M-TP and MK performed the experiments and wrote the scripts; M-TP, MK, and PS validated the analysis; and GP provided funding. All authors wrote and approved the manuscript.

## Conflict of Interest

GPP is Full Member and National Representative at the European Medicines Agency, Committee for Human Medicinal Products (CHMP)—Pharmacogenomics Working Party in Amsterdam, the Netherlands.

The remaining authors declare that the research was conducted in the absence of any commercial or financial relationships that could be construed as a potential conflict of interest.
