# Supplementary material for: A Novel Text-Mining Approach for Retrieving Pharmacogenomics Associations From the Literature
Source: Front Pharmacol. 2020 Nov 10;11:602030. doi: 10.3389/fphar.2020.602030 (PMC7748107; doi:10.3389/fphar.2020.602030)
Supplement: Supplementary file 1 [file datasheet1.docx]

**Supplemental material**

**Table S1: Confusion matrix for a binary classification task.** A confusion matrix contains 4 basic parameters, named True Positive (TP), False Positive (FP), True Negative (TN) and False Negative (FN) values. The term “Positive” represents the class of interest in a specific classification task and in this case, it refers to sentences classified as “Correlated”. In a binary classification problem (i.e. one where the instances can be classified in one of two possible classes), those four parameters represent: the instances that belong to the class of interest (Positive) and are classified by the model as such (TP); the instances that belong to the remaining class (Negative) but are classified by the model as Positive (FP); the instances that belong to the remaining class (Negative) and are classified by the model as such (TN); the instances that belong to the class of interest (Positive) but are classified by the model as Negative (FN). In the case of multiclass classification problems (i.e. for n-pair sentences), alternative approaches can be followed for the determination of TP, TN, FP and FN, such as the one-vs-all approach, where the Negative class consists from the combination of any other class than the one of interest (Positive).

|  | **Prediction** | | |
| --- | --- | --- | --- |
| **Reality** |  | **Positive class** | **Negative class** |
|  | **Positive class** | TP | FN  Type II error |
|  | **Negative class** | FP  Type I error | TN |

**Table S2: Mathematic formulas and explanation of the evaluated performance metrics**

| **Metric** | **Mathematic formula** | **Explanation** |
| --- | --- | --- |
| Accuracy | $\frac{TP+TN}{TP+FP+TN+FN}$ | Evaluation of the overall performance of the classifier |
| Sensitivity (= Recall) | $\frac{TP}{TP+FN}$ | The classifier’s effectiveness on identifying data points belonging to the class of interest (Positive) |
| Specificity | $\frac{TN}{FP+TN}$ | The classifier’s effectiveness on identifying data points belonging to the remaining class (Negative) |
| Prevalence | $\frac{TP+FN}{TP+FP+TN+FN}$ | The portion of the data points that belong to the Positive class |
| Precision | $\frac{TP}{TP+FP}$ | The portion of the data points that were classified as Positive that belong to this class |
| Positive Predictive Value (PPV) (=Precision) | $\frac{Sensitivity*Prevalence}{Sensitivity*Prevalence+\left( 1-Sensitivity \right)*(1-Prevalence)}$ | The portion of the data points that were classified as Positive that belong to this class |

| Negative Predictive Value (NPV) | $\frac{Specificity*(1-Prevalence)}{\left( 1-Sensitivity \right)*Prevalence+Specificity*(1-Prevalence)}$ | The portion of the data points that were classified as Negative that belong to this class |
| --- | --- | --- |
| Detection Rate | $\frac{TP}{TP+FP+TN+FN}$ | The portion of the truly positive data points compared to all instances |
| Detection Prevalence | $\frac{TP+FP}{TP+FP+TN+FN}$ | The portion of the classified as positive data points compared to all instances |
| F1 | $\frac{\left( 1+ {beta}^{2} \right)*Precision*Recall}{{beta}^{2}*Precision+Recall}, beta=1$ | Harmonic mean of precision and recall |
| Balanced Accuracy | $\frac{Sensitivity+Specificity}{2}$ | The mean of sensitivity and specificity |

**Table S3: Mean accuracy with lower and upper ranger for all models trained using the two sets of training sentences (1-pair and n-pair).**

| **Classifier** | **1 pair** | **n pair** |
| --- | --- | --- |
| **FastText** | 0.72 [0.61, 0.82] | 0.5 [0.39, 0.61] |
| **Linear SVM** | 0.74 [0.63, 0.84] | 0.56 [0.45, 0.67] |
| **XGBoost** | 0.75 [0.63, 0.84] | 0.62 [0.52, 0.73] |
| **glmnet** | 0.71 [0.62, 0.83] | - 1. [0.49, 0.7] |

**List of custom defined words** **that was used to filter the training and the unseen sentences**

The sentences used for training were filtered to maintain at least one of the following words (or parts of words).

(*) means "anything" (eg, associat* means we can get words containing 'associat' and anything after that, like associated, association etc)

(^) means "starting with" (eg ^find means that the term "find" must be in the beggining of a word, and not to be inside a word- the word nees to start with "find...")

($) means "ending with" (the term must be found in the end of a word, for example in order to get "biomarker" and "marker" at the same time, we can use "*marker$")

^associat*

^modulat*

^influenc*

^alter*

^affect*

^correlat*

^significan*

^contributor*

^predictive*

^increas*

^decreas*

^reduc*

^interact*

^impact*

^response*

^tolerance*

^resistan*

*marker$

*markers$

^toxicity*

^adverse*

^AE$

^ADR$

^ADRs$

^AEs$
